# Reducing and controlling metabolic active tumor volume prior to CAR T-cell infusion can improve survival outcomes in patients with large B-cell lymphoma

**DOI:** 10.1038/s41408-024-01022-w

**Published:** 2024-03-07

**Authors:** Kylie Keijzer, Janneke W. de Boer, Jaap A. van Doesum, Walter Noordzij, Gerwin A. Huls, Lisanne V. van Dijk, Tom van Meerten, Anne G. H. Niezink

**Affiliations:** 1 Department of Hematology, University of Groningen, University Medical Center Groningen, Hanzeplein 1, 9713GZ Groningen, The Netherlands; 2 Department of Radiation Oncology, University of Groningen, University Medical Center Groningen, Hanzeplein 1, 9713GZ Groningen, The Netherlands; 3 Department of Nuclear Medicine and Molecular Imaging, University of Groningen, University Medical Center Groningen, Hanzeplein 1, 9713GZ Groningen, The Netherlands

**Keywords:** B-cell lymphoma, B-cell lymphoma, Medical imaging, Immunotherapy

## Abstract

Bridging therapy before CD19-directed chimeric antigen receptor (CAR) T-cell infusion is frequently applied in patients with relapsed or refractory Large B-cell lymphoma (r/r LBCL). This study aimed to assess the influence of quantified MATV and MATV-dynamics, between pre-apheresis (baseline) and pre-lymphodepleting chemotherapy (pre-LD) MATV, on CAR T-cell outcomes and toxicities in patients with r/r LBCL. MATVs were calculated semi-automatically at baseline (*n* = 74) and pre-LD (*n* = 68) in patients with r/r LBCL who received axicabtagene ciloleucel. At baseline, patients with a low MATV (< 190 cc) had a better time to progression (TTP) and overall survival (OS) compared to high MATV patients (*p* < 0.001). High MATV patients who remained stable or reduced upon bridging therapy showed a significant improvement in TTP (*p* = 0.041) and OS (*p* = 0.015), compared to patients with a high pre-LD MATV (> 480 cc). Furthermore, high MATV baseline was associated with severe cytokine release syndrome (CRS, *p* = 0.001). In conclusion, patients with low baseline MATV had the best TTP/OS and effective reduction or controlling MATV during bridging improved survival outcomes in patients with a high baseline MATV, providing rationale for the use of more aggressive bridging regimens.

## Introduction

CD19-targeted chimeric antigen receptor (CAR) T-cell therapy significantly improved outcomes compared to standard 2nd and 3rd line immunochemotherapy in patients with relapsed or refractory Large B-cell Lymphoma (r/r LBCL) [1–3]. In this strategy, autologous T-cells are genetically engineered to express a CAR receptor that targets CD19-antigens expressed on tumor cells and are subsequently expanded in vitro. Upon testing for quantity, quality and sterility, and after the patient received lymphodepleting chemotherapy, the final product is administered in a single infusion. As this manufacturing process is complex, the total time between setting the indication for CAR T-cell therapy and the actual CAR T-cell infusion may take up to 4–6 weeks. Therefore, many patients might require bridging therapy in between apheresis and CAR T-cell infusion in order to limit disease progression, diminish lymphoma-related symptoms and/or reduce tumor volume.

Bridging therapy may include systemic therapy (e.g., chemotherapy, targeted therapy or steroids), radiotherapy or a combination of both modalities. Bridging therapy has shown to be feasible, safe and effective [4]. Moreover, no significant differences in severe CAR T-cell related toxicity have been observed between different bridging modalities and patients who did not receive bridging [4, 5]. Response to bridging might lead to a reduction in risk of progressive disease, death and serious (grade 3–4) immune effector cell-associated neurotoxicity syndrome (ICANS) [4, 5]. In order to more precisely characterize this response to bridging, it is essential to define the reduction in tumor volume resulting from the different bridging modalities in relation to CAR T-cell therapy outcomes.

The measurement of metabolic active tumor volume (MATV) using ^18^F-Fluorodeoxyglucose Positron Emission Tomography/Computerized Tomography (^18^F-FDG PET/CT) scans provides a quantification of tumor volume. MATV is a measure of disease burden and indicates the total metabolically active tumor mass throughout the body. Previous studies have shown that patients with a high MATV before CAR T-cell infusion have inferior outcome (HRs ranging from 1.27 to 5.27) [6–9] and are also more likely to develop toxicity [6, 10]. Until now, only one study evaluated paired pre-apheresis and pre-lymphodepleting chemotherapy (pre-LD) scans, showing that this captures important information, as an increase in extranodal MATV from pre-apheresis to pre-LD scans is associated with an increased risk of death [11]. However, detailed information about MATV dynamics caused by different bridging strategies is still lacking.

Unlike other pretreatment factors associated with CAR T-cell therapy outcomes, such as elevated lactate dehydrogenase (LDH), bulky disease, or involvement of ≥3 extranodal localizations [12–14], MATV determined at baseline and pre-LD can capture precise and objective information regarding the efficacy of the applied bridging therapy. Therefore, this study aims to investigate the relation between MATV and MATV-dynamics (baseline to pre-LD) with CAR T-cell therapy outcomes and toxicities, including the influence of different bridging strategies on MATV and CAR T-cell outcomes in patients with r/r LBCL.

## Materials and methods

### Patient population

All LBCL patients including Diffuse large B-cell lymphoma (DLBCL), transformed follicular lymphoma (tFL) and High-grade B-cell lymphoma (HGBCL), not otherwise specified (HGBCL NOS) or double/triple hit (HGBCL DH/TH) treated with axicabtagene ciloleucel (axi-cel) between October 2017 and September 2022 were consecutively included in this study. Patients participated in either a clinical trial (ZUMA-1 [NCT02348216], ZUMA-7 [NCT03391466]), an early access program, or received axi-cel as standard of care, and received CAR T-cell therapy as a second- or third-line treatment. Only patients that received CAR T-cell infusion were included. Patients with central nervous system (CNS) localizations were excluded. Prospective data were available on patient, tumor and outcome characteristics. ^18^F-FDG-PET scans were performed at baseline (before apheresis) and pre-LD (after bridging therapy, before start lymphodepleting chemotherapy). This study was carried out in accordance with the applicable rules concerning the review of research ethics committee and patients gave informed consent for OncoLifeS registration [15].

### Bridging strategies

Bridging therapy was defined as any therapy targeting LBCL administered between apheresis and lymphodepleting chemotherapy. If pre-apheresis therapy was administered, it was defined as bridging therapy only if it was given after baseline ^18^F-FDG-PET imaging. Based on individual patient characteristics: tumor volume, tumor location, disease progression and response to earlier lines of treatment, the type of bridging was chosen. Applied bridging strategies consisted of focal radiotherapy, systemic therapy and a combination of both. Radiotherapy was mostly given in 5 to 15 fractions, with a total dose of 20−30 Gy. Systemic therapy included steroids, one cycle of immunochemotherapy (e.g., rituximab-gemcitabine oxaliplatin, rituximab-polatuzumab-vedotin-bendamustine) and single agent immunotherapy (e.g., single dose rituximab). Combination therapy included a combination of both radiotherapy and systemic therapy regimens.

### Image acquisition and MATV measurement

Baseline ^18^F-FDG PET/CT scans were performed at either the UMCG or at referral centers, and pre-LD scans were always performed at the UMCG (Supplementary File 1). Scans were analyzed using EARL1 accredited reconstructions according to the European Association of Nuclear Medicine (EANM) [16, 17] and were acquired from the skull to mid-thigh or full-body, depending on which was available. MATVs were extracted using the MUST-segmenter [18], from both baseline and pre-LD ^18^F-FDG-PET scans in a semi-automatic manner. To avoid underestimation of MATV and maintain robustness, a Standardized Uptake Value (SUV) threshold of 2.5 was chosen (SUV2.5) [11, 18–20]. Both nodal and extranodal sites were included; spleen and bone marrow involvement were incorporated only if there was focal uptake [21]. All tumors were visually inspected to ensure that only pathological lesions were incorporated. If necessary, delineation was multidisciplinary discussed until consensus was reached.

### Definitions and endpoints

The primary endpoints of the study were Time to Progression (TTP), defined as time from CAR T-cell apheresis to disease progression, and overall survival (OS), defined as the time from CAR T-cell apheresis to death from any cause. Survival was truncated at 2 years, since the relevant events occurred within this timeframe. TTP was preferred over progression-free survival to evaluate the relation between MATV and progression after CAR-T, as it excludes non-relapse related mortality. Response evaluations prior to and after CAR T-cell therapy were defined with ^18^F-FDG-PET assessments using the Lugano 2014 criteria [22]. Overall response rate (ORR) was determined as a complete response (CR) or partial response (PR) to therapy. Secondary endpoints consisted of ≥ grade 2 Cytokine Release Syndrome (CRS) and ICANS, graded according to the American Society for Transplantation and Cellular Therapy (ASTCT) criteria [23]. Both CRS and ICANS events were defined as a grade ≥ 2, given its clinically relevant nature necessitating close monitoring and medical intervention. In addition, it ensures sufficient power of the analysis.

### Statistical analyses

Descriptive statistics of variables were provided, including median with interquartile range (IQR). The Wilcoxon signed-rank test was used to compare baseline MATVs and pre-LD MATVs, and the Mann-Whitney U test was used to compare MATVs between patient groups. The optimal MATV cut-off to predict TTP at 2 years was identified by maximizing the Youden index of the Receiver Operator Characteristic (ROC) curve (sensitivity + specificity − 1). The Kaplan-Meier method was applied to estimate 2-year TTP and OS rates, and the log-rank test was utilized to compare the differences among patient groups. Univariable analysis was performed with Cox regression for TTP and OS, and with logistic regression for CRS and ICANS. Hazard- and odd ratios, including the 95% confidence intervals (CI), were reported. *P* < 0.05 was considered statistically significant. Statistical analyses were performed using R v4.2.1.

## Results

### Patient and treatment characteristics

Seventy-four patients with r/r LBCL who were treated with axi-cel between October 2017 and September 2022 in the University Medical Center Groningen (UMCG), were included in this study (Table 1). During this period, 7 patients underwent apheresis without proceeding to CAR T-cell infusion. Reasons for drop-out were heterogeneous and consisted of progressive disease (*n* = 3), failure to meet criteria for infusion (*n* = 2), death due to hemorrhagic cerebrovascular accident (*n* = 1) and production failure of the CAR T-cell product (*n* = 1).

**Table 1:** Patient, treatment and outcome characteristics.

Characteristic	Total (*n* = 74)
Age in years, median (range)	61 (20–79)
Age > 65 years, *n* (%)	24 (32.4)
Gender, male, *n* (%)	51 (68.9)
Lymphoma histology, *n* (%)	
DLBCL	40 (54.1)
tFL	19 (25.7)
HGBCL DH/TH	10 (13.5)
HGBCL NOS	5 (6.8)
ECOG PS, *n* (%)	
0	55 (74.3)
1	17 (23.0)
2	2 (2.7)
Disease stage (at baseline), *n* (%)	
Stage I−II	12 (16.2)
Stage III−IV	62 (83.8)
Nr. of extranodal sites, n (%)	
0	23 (31.1)
1	32 (43.2)
≥2	19 (25.7)
LDH at screening > ULN^a^, *n* (%)	42 (56.8)
Missing	2 (2.7)
LDH pre-infusion > ULN^a^, *n* (%)	24 (32.4)
IPI, *n* (%)	
Low	15 (20.3)
Low-intermediate	26 (35.1)
High-intermediate	27 (36.5)
High	4 (5.4)
Missing	2 (2.7)
Previous lines of therapy, median (range)	2 (1–6)
Primary refractory first line, *n* (%)	51 (68.9)
Primary refractory second line, *n* (%)	53 (71.6)
N/A	10 (13.5)
Days between apheresis and infusion, median (IQR)	31 (28–34)
Bridging therapy, *n* (%)	
No bridging	21 (28.4)
Radiotherapy	31 (41.9)
Systemic therapy	11 (14.9)
Combination	11 (14.9)
Clinical response to CAR T-cell therapy, *n* (%)	
CR by last follow-up	38 (51.4)
Best ORR	64 (86.5)
CRS grade, *n* (%)	
No CRS	12 (16.2)
1	34 (45.9)
2	26 (35.1)
3	2 (2.7)
ICANS grade, *n* (%)	
No ICANS	38 (51.4)
1	12 (16.2)
2	12 (16.2)
3	10 (13.5)
4	2 (2.7)

*CAR T-cell therapy* chimeric antigen T-cell therapy, *CR* Complete Response, *CRS* cytokine release syndrome, *ICANS* immune effector cell-associated neurotoxicity syndrome, *DLBCL* Diffuse Large B-cell lymphoma, *ECOG PS* Eastern Cooperative Oncology Group performance status, *HGBCL* *DH/TH* high-grade B-cell lymphoma double-hit/triple-hit, *HGBCL NOS* high-grade B-cell lymphoma not otherwise specified, *IPI* International Prognostic Index, *IQR* interquartile range, *N/A* not applicable, *LDH* lactate dehydrogenase, *ORR* Overall Response Rate, *tFL* transformed follicular lymphoma.

^a^ULN = 248 for males and 247 for females.

Age ranged from 20 to 79 years (median: 61). Most patients had stage III/IV disease (83.8%), involvement of extranodal sites (68.9%), and an ECOG performance status of 0–1 (97.3%). IPI at baseline was high-intermediate in 27 (36.5%) patients and high in 4 (5.4%) patients. Median LDH at screening was 275 U/L (IQR 218–403) and at the time of infusion 219 U/L (IQR 176–258). The majority of patients had chemotherapy refractory disease as they were primary refractory to first line therapy (68.9%), as well as to second line therapy (71.6%).

Most patients received CAR T-cell therapy as a third-line treatment (93.2%). The median time between apheresis and CAR T-cell infusion was 31 days. Bridging therapy was given to 53 (71.6%) patients, where radiotherapy was most frequently applied (*n* = 31; 41.9%). Eleven patients (14.9%) received systemic therapy and 11 patients (14.9%) received combination therapy. The overall response rate (ORR) to CAR T-cell therapy was 56.8% and the median follow-up since apheresis was 12.0 months. The best ORR was 86.5%, whereof 73.0% CR and 13.5% PR; for 13.5% of the patients progressive disease was the best response. After CAR T-cell infusion, disease progression was observed in 21 patients and 22 patients died. In the majority of cases, reason of death was progressive disease or relapse after CAR T-cell infusion (*n* = 21). One patient died due to severe ICANS. Both median TTP and OS were not reached, with a 2-year TTP rate of 71.6% and 2-year OS rate of 70.3%. Any CRS grade was observed in 62 patients (83.8%) and 28 patients (37.8%) had a grade ≥ 2 CRS, whereas only 2 patients (2.7%) had grade ≥ 3 CRS. Thirty-six patients (48.6%) experienced ICANS of which 24 patients (32.4%) had a grade ≥ 2 ICANS and 12 patients (16.2%) grade ≥ 3 ICANS.

### MATV characteristics between baseline and pre-lymphodepleting chemotherapy

**Fig. 1:**
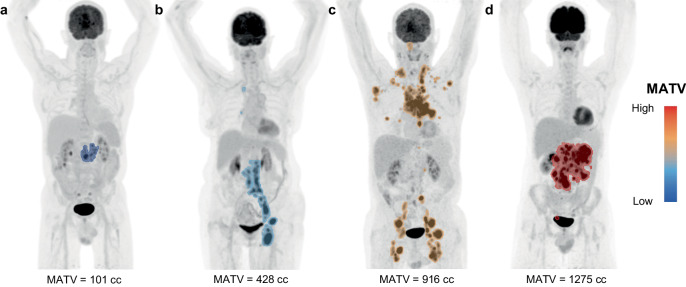
Maximum Intensity Projections (MIPs) and segmentations of 4 patients with different MATVs. Examples of corresponding segmentations are depicted as an overlay on the MIPs, going from low (**a**), to low-intermediate (**b**), to intermediate-high (**c**), to high (**d**) MATV.

The median time between baseline ^18^F-FDG-PET and apheresis was 12 days [IQR 6–19 days], whereas the median time between pre-LD ^18^F-FDG-PET and infusion was 6 days [IQR 6–7 days]. Baseline ^18^F-FDG-PET scans were available for all patients (*n* = 74). For 6 patients (5 of which were treated in a clinical trial) no pre-LD scan was performed, resulting in 68 available pre-LD scans; due to logistic or patient-related reasons, 7 patients started lymphodepleting chemotherapy after performing the pre-LD scan. Segmentations and MATV values of patients with varying amounts of disease are depicted in Fig. 1, illustrating the visual differences between low and high MATV values.

**Fig. 2:**
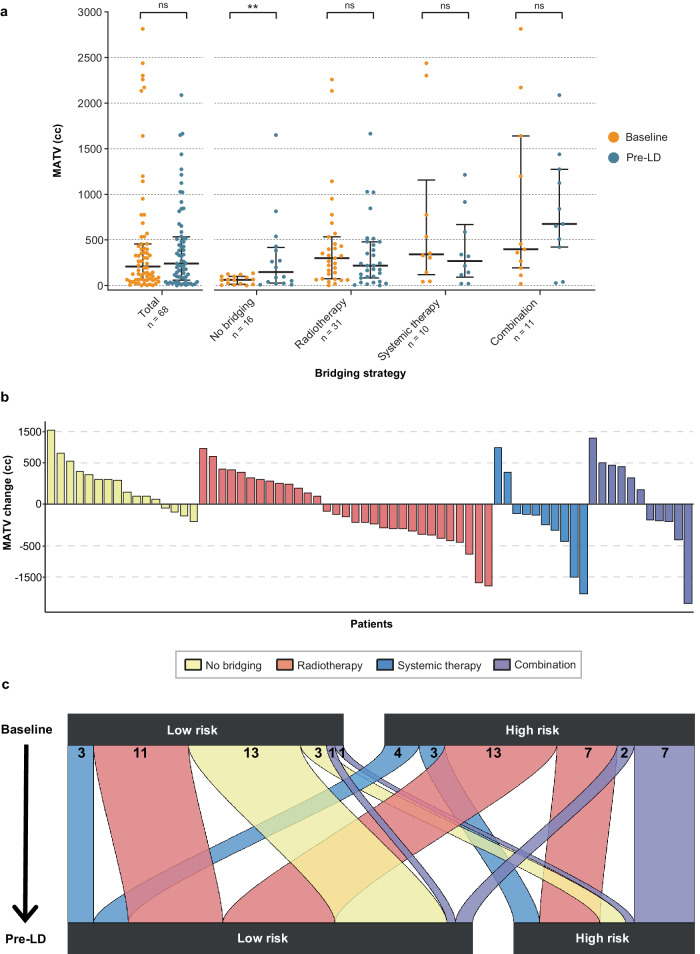
Total MATV information regarding applied bridging strategies between baseline and pre-LD (*n* = 68). Distributions of MATVs available at both baseline and pre-LD, also categorized by applied bridging strategy (**a**). The crossbars depict the median and the whiskers depict the IQR. Absolute MATV change per patient between baseline and pre-LD (**b**), colored by bridging strategy. Number of patients per MATV risk group and bridging strategy group, as they transfer from baseline to pre-LD (**c**).

Median baseline MATV of the full cohort was 180 cc [IQR 64–426] (*n* = 74). At pre-LD, the median MATV increased to 241 cc [IQR 75–525] but was not significantly different from baseline (*p* = 0.795; *n* = 68; Fig. 2a). Patients who received no bridging therapy showed an increase in MATV (*p* = 0.008), where the median MATV baseline was 62 cc [IQR 18–100] and changed to 147 cc [IQR 36–395] at pre-LD. Patients who received radiotherapy showed a non-significant MATV decrease; the median MATV baseline of 300 cc [IQR 101–495] decreased to a median of 217 cc [IQR 89–462] at pre-LD (*p* = 0.193). This also holds for patients who received systemic therapy, with a median MATV baseline of 341 cc [IQR 184–715] and a median MATV pre-LD of 268 cc [IQR 123–524] (*p* = 0.193). In contrast, for patients who underwent combination therapy (*p* = 0.577) a non-significant increase was observed, with a median MATV baseline of 398 cc [IQR 230–1420] and a median MATV pre-LD of 675 cc [IQR 466–1199].

The individual bridging impact on MATV is shown in Fig. 2b. For patients who were bridged with radiotherapy, a decrease of MATV was predominantly seen; an increase of MATV was observed when a patient had outfield progression. Systemic therapy and combination therapy showed a more mixed response, while patients where no bridging therapy was applied showed an overall increase (hence progression), in concordance with Fig. 2a.

### Association of MATV with time to progression (TTP) and overall survival (OS)

**Fig. 3:**
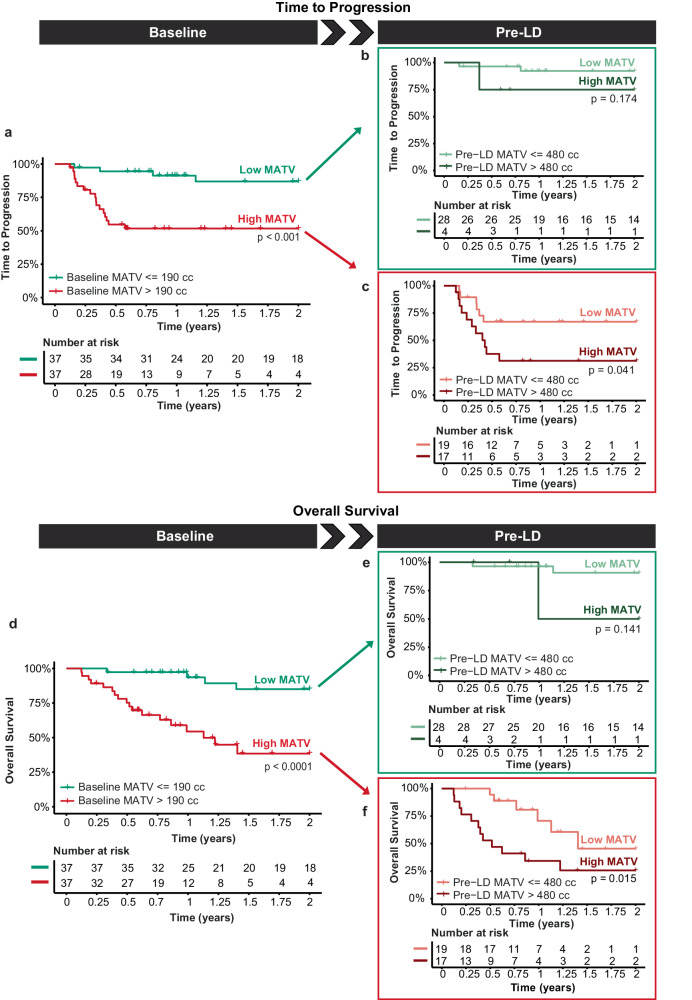
Survival outcomes in patients with low- or high-MATV at baseline and pre-LD. Kaplan Meier estimates of 2-year TTP and OS of patients with low- or high-MATV at baseline (*n* = 74; **a**, **d**) and further distinguished into low- and high-MATV at pre-LD (*n* = 68; **b**, **c**, **e**, **f**; Supplementary Fig. 1).

The optimal MATV cut-off points for 2-year TTP at baseline and pre-LD were 190 cc and 480 cc, respectively. At baseline, low-MATV patients had a significant better TTP of 86.9% (*p* < 0.001), compared to high-MATV patients with a TTP of 51.7% (Fig. 3a). The same was observed for OS (Fig. 3d), with 85.0% OS for low-MATV patients compared to 38.5% OS for high-MATV patients (*p* < 0.0001).

After the bridging period, at pre-LD, a significant improvement (*p* = 0.041) was observed in TTP between patients who initially had a high MATV at baseline (Fig. 3a lower line) and were successfully bridged to a low MATV (67.1% survival; Fig. 3c upper line), compared to those who maintained a high MATV throughout the bridging course (31.4% survival; Fig. 3c lower line). A similar trend was observed for OS (Fig. 3f; *p* = 0.015). For these patients with both a high MATV at baseline and pre-LD, the median TTP and OS were reduced to 5.1 months and 6.2 months, and a 2-year OS rate of 31.4% and 25.7%, respectively. This shows that effective control of MATV by bridging therapy for patients who have an initial high-MATV baseline is associated with improved TTP and OS rates.

Patients with low MATV at baseline (Fig. 3a, d upper line) that remained low at pre-LD showed the best TTP (92.2%; Fig. 3b upper line) and OS (90.8%; Fig. 3e upper line). Yet, for the low-MATV patients at baseline that progressed to have a high MATV at pre-LD, poorer TTP rates (75.0%; Fig. 3b lower line) and OS rates (50.0%; Fig. 3e lower line) were observed, but with only 4 patients in this group this difference was not significant (*p* > 0.1).

### Bridging strategies in the high- and low-MATV patient groups

To further investigate the applied bridging strategies in the MATV patient groups, the risk paths of patients from baseline to pre-LD are depicted in Fig. 2c. For patients who were bridged with radiotherapy and had a high-MATV baseline, the majority transitioned to low-MATV at pre-LD (*n* = 13). Additionally, low-MATV baseline patients who received radiotherapy remained low at pre-LD (*n* = 11), which also applies for most patients who did not undergo any bridging therapy (*n* = 13). Furthermore, as mentioned before, systemic- and combination therapy show mixed results.

### Patient characteristics in the high- and low-MATV patient groups

Patient, treatment and outcome characteristics per MATV risk group are described in Supplementary Table 3. LDH at screening and pre-infusion were both different among the 4 risk groups (*p* = 0.002), with the largest median LDH in the highest risk group (median LDH at screening 451 [IQR 290−635]; median LDH pre-infusion 315 [IQR 226−484]). Furthermore, gender, follow-up time and best ORR differed between the risk groups. Other patient and treatment characteristics did not significantly differ between the risk groups, including age, IPI-score, number of extranodal sites, disease stage, ECOG performance status and primary refractory to first/second line treatment.

### Association of MATV with toxicity

**Fig. 4:**
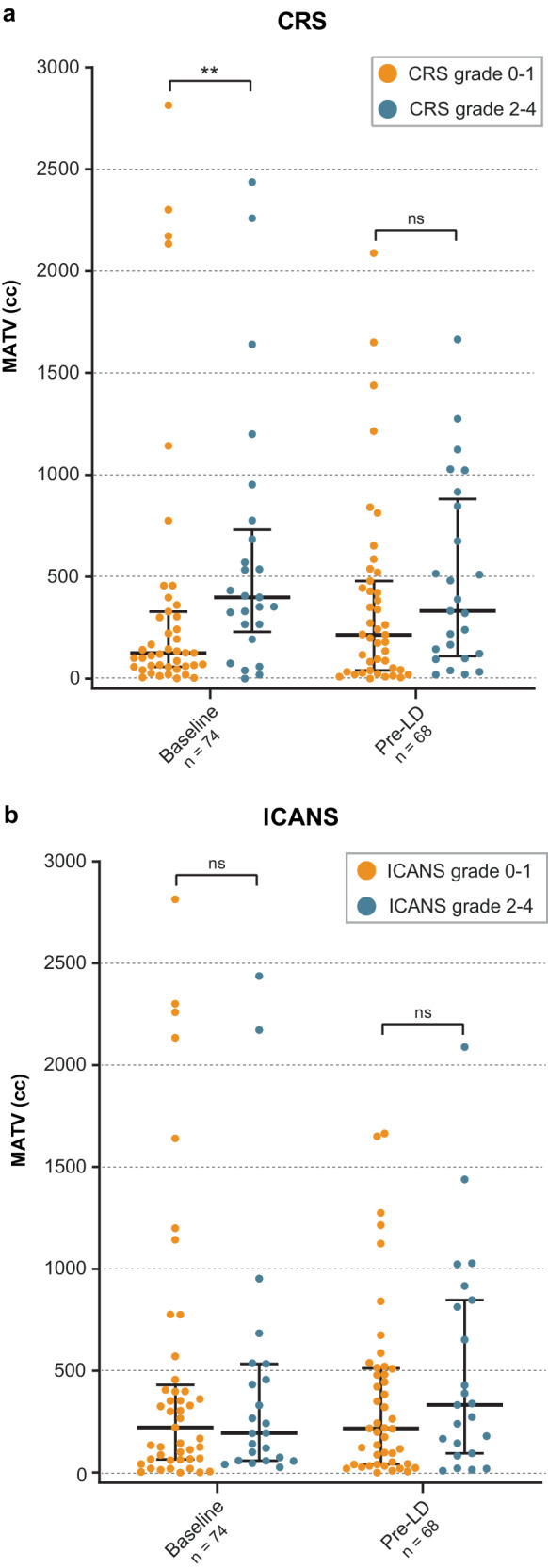
MATVs at baseline (*n* = 74) and pre-LD (*n* = 68) categorized by toxicity incidence. Toxicity grades 0–1 represented in orange and grades 2–4 represented in turquoise for CRS (**a**) and ICANS (**b**). The crossbars depict the median and the whiskers depict the IQR.

Baseline MATV was significantly higher (*p* = 0.008) in patients with CRS ≥ 2 (median: 398 cc [IQR 266–684]), compared to patients with a grade 0–1 CRS (median: 125 cc [IQR 60–317]) (Fig. 4a). The median pre-LD MATV was also higher in patients with CRS ≥ 2 332 cc [IQR 123–846], compared to patients with a grade 0–1 CRS (215 cc [IQR 41–462]), yet this was not a significant difference (*p* = 0.131). For patients with ICANS ≥ 2 the median baseline MATV (194 cc [IQR 67–495]) was lower, compared to ICANS grade 0–1 (222 cc [IQR 66–406]; *p* = 0.827). This was higher for MATV pre-LD (332 cc [IQR 120–830]) compared to ICANS 0–1 (217 cc [IQR 43–510]; *p* = 0.374) (Fig. 4b). However, both these differences were not significant.

### Univariable analysis for survival and toxicity

Univariable Cox regression analysis demonstrated that a high baseline MATV was associated with a worse TTP compared to a low baseline MATV (HR 5.97 CI [2.00–17.80]; *p* = 0.001; Table 2), indicating a decreased prognosis for patients with larger tumor volumes. A similar association was observed between a high MATV baseline and OS (HR 6.90 CI [2.33–20.70]; *p* < 0.001). In addition, high MATV pre-LD had a comparable relation with TTP (HR 4.56 CI [1.85–11.20]; *p* < 0.001) and OS (HR 5.80 CI [2.39–14.10]; *p* < 0.001). Other markers that were significantly associated with TTP and OS were found for pre-LD LDH (> ULN), gender (for OS only) and bridging therapy (for OS only).

**Table 2:** Univariable analysis for TTP, OS, CRS and ICANS.

	TTP		OS		CRS		ICANS	
	HR [95% CI]	*P* value	HR [95% CI]	*P* value	OR [95% CI]	*P* value	OR [95% CI]	*P* value
Gender (male)	3.17 [0.93−10.80]	0.065	3.80 [1.13−13.00]	**0.032**	0.92 [0.34−2.54]	0.878	0.49 [0.18−1.38]	0.176
Age (> 65 years)	0.47 [0.16−1.39]	0.172	0.63 [0.23−1.70]	0.358	1.64 [0.61−4.44]	0.328	1.84 [0.66−5.09]	0.243
Stage (III–IV)	2.26 [0.53−9.70]	0.274	2.50 [0.58−10.60]	0.224	0.83 [0.23–2.91]	0.765	0.62 [0.17−2.20]	0.458
IPI (≥ 3)	0.61 [0.23−1.58]	0.308	0.72 [0.29−1.78]	0.471	0.61 [0.23−1.61]	0.317	0.79 [0.29−2.16]	0.645
Extranodal sites (yes vs no)	2.21 [0.74−6.58]	0.153	2.20 [0.74−6.45]	0.158	0.92 [0.34−2.54]	0.878	4.67 [1.23−17.74]	**0.024**
Bridging therapy (yes vs no)	3.12 [0.92−10.60]	0.068	3.50 [1.02−11.70]	**0.046**	3.52 [1.04−11.87]	**0.043**	1.29 [0.43−3.88]	0.656
High baseline MATV (> 190 cc)	5.97 [2.00−17.80]	**0.001**	6.90 [2.33−20.70]	**<** **0.001**	5.63 [1.97−16.05]	**0.001**	1.28 [0.48−3.40]	0.620
High pre-LD MATV (> 480 cc)	4.56 [1.85−11.20]	**<** **0.001**	5.80 [2.39−14.10]	**<** **0.001**	2.59 [0.90−7.49]	0.078	1.31 [0.45−3.84]	0.619
Elevated LDH at screening (> ULN)	0.74 [0.31−1.78]	0.504	0.85 [0.36−1.99]	0.703	0.92 [0.35−2.41]	0.870	0.69 [0.25−1.88]	0.469
Elevated LDH pre-LD (> ULN)	2.51 [1.06−5.94]	**0.036**	3.30 [1.42−7.73]	**0.005**	2.13 [0.78−5.76]	0.138	1.40 [0.50−3.90]	0.520

*CI* confidence intervals, *HR* hazard ratios, *CRS* cytokine release syndrome, *ICANS* immune effector cell-associated neurotoxicity, *IPI* International Prognostic Index, *LDH* lactate dehydrogenase, *MATV* metabolic activated tumor volume, *OR* odd ratios, *OS* overall survival, *pre-LD* pre-lymphodepleting therapy, *TTP* Time to Progression, *ULN* upper limit of normal.

Bold values indicates statistical significant *p*-value < 0.05.

Development of CRS ≥ 2 was significantly associated with a high baseline MATV (OR 5.63 CI [1.97–16.05]; *p* = 0.001) and bridging therapy (OR 3.52 CI [1.04–11.87]; *p* = 0.043). Development of ICANS ≥ 2 was only significantly associated with extranodal sites (OR 4.67 CI [1.23–17.74]; *p* = 0.024). An univariable analysis of additional parameters is provided in Supplementary Table 1.

## Discussion

We report on the impact of MATV and MATV-dynamics during the bridging period upon CAR T-cell treatment in patients with r/r LBCL who were treated with axi-cel at a single institution.

In this analysis, patients with a low baseline MATV had the best TTP and OS. In addition, patients with an initial high baseline MATV, but in which tumor volume could be reduced or controlled during the bridging period, had a better outcome compared to patients for whom bridging therapy did not lead to tumor reduction.

Nowadays, in real-world, a significant number of patients receive bridging therapy prior to CAR T-cell infusion [12–14]. Patients responding to bridging therapy, regardless of the applied regimen, had a reduced risk of progression after CAR T-cell infusion [4]. In addition, others have found that a higher MATV determined prior to CAR T-cell infusion is associated with disease progression after CAR T-cell therapy [11, 24–26]. Breen et al. discovered that especially an increased tumor volume from apheresis to pre-LD is associated with an increased risk of death [11]. These results align with our findings that effective reduction or control of tumor volume results in an improved survival.

Of note, it is difficult to distinguish the effect of disease reduction from underlying differences in disease biology to establish these results. Patients with a high baseline MATV that showed effective control of tumor volume during the bridging period could represent a subset of patients that is more sensitive to bridging therapy and CAR T-cell therapy, caused by a preferable, less aggressive disease biology. However, in our analysis we did not find any significant differences in the distribution of well-known markers of aggressive disease, such as lymphoma histology, advanced stage, a high number of extranodal sites and a high IPI-score between these subgroups. Moreover, no significant differences were observed in the percentage of patients that were primary refractory to first and second line immunochemotherapy between the different subgroups. Altogether, despite the unknown molecular composition of the tumor itself, these data suggest that disease reduction in patients with a high disease burden at baseline has a positive effect on CAR T-cell outcome, pointing to an opportunity for further exploration of the implementation of more effective (aggressive) bridging regimens.

Furthermore, in our cohort radiotherapy was chosen in the majority of patients in need of bridging therapy. Radiotherapy was highly effective as a single-modality bridging regimen in controlling MATV in patients with a low-risk MATV at baseline. In addition, patients bridged with radiotherapy were most likely to shift from the high-risk MATV at baseline to the low-risk group at pre-LD. Both of these findings are in line with the previously found advantages of radiotherapy bridging for patients with a high tumor load [27]. This highlights the potential of radiotherapy as a bridging strategy in this heavily pretreated patient group.

In our cohort, baseline and pre-LD ^18^F-FDG PET/CT-scans were conducted with limited deviation in timing from apheresis and infusion. Uniquely, these scans were performed irrespective of the application of bridging therapy or clinical indication for progression. Therefore, we were able to provide MATV-dynamics of both patients who did and did not receive bridging therapy. It is known that patients in need of bridging therapy during this period are more likely to have a high IPI-score, elevated LDH and bulky disease, and have a worse outcome after CAR T-cell infusion compared to the patients without an apparent need for bridging therapy [5]. In alignment, in our cohort patients with a high baseline MATV had indeed a higher LDH (Supplementary Table 2), suggesting more aggressive disease.

Even though most patients who did not undergo bridging therapy showed an increase in MATV, only a few patients transitioned from low-risk at baseline to high-risk at pre-LD (Fig. 2). Moreover, our results showed a clear survival benefit for patients who start with low baseline MATV, as reported previously [5]. This emphasizes the importance of rapid screening, apheresis and treatment if patients have an indication for CAR T-cell therapy.

Moreover, we found that a high MATV baseline (> 190 cc) was associated with a higher risk of CRS grade ≥ 2. Other studies have found similar results for CRS grade ≥ 3 [6, 10]. Due to low incidence of CRS grade ≥ 3 in our cohort, this association was not evaluated. On the contrary, MATV at pre-LD was not associated with CRS grade ≥ 2. Data regarding pre-LD MATV and CRS are conflicting [24, 26]. Similarly, inconsistent findings for the relation of pre-LD MATV with ICANS are reported [11, 26, 28]. We observed no association of both baseline and pre-LD MATV with ICANS grade ≥ 2.

In our cohort, median MATV increased from baseline to pre-LD in general, indicating the tendency for progressive disease in these patients despite the bridging strategies that were applied in the majority of patients. The optimal cut-off values differed between baseline and pre-LD. This can partly be explained by the median increase of MATV from baseline to pre-LD and fast progressive disease in patients not responding to bridging therapy. On the other hand, radiotherapy is most commonly used within our cohort, which is a local treatment and disease progression is expected outside the radiotherapy treatment field. Also, response assessment after radiotherapy with ^18^F-FDG PET/CT-scan was performed after less than 2 weeks. Normally, response assessment is performed after more than 6 weeks, suggesting that the effect of radiotherapy might be underestimated.

We found optimal cut-off values of 190 cc at baseline and 480 cc at pre-LD. This is substantially higher compared to other studies. The studies of Dean et al., Galtier et al., Hong et al. and Iacoboni et al. reported optimal cut-offs of 147 cc [6], 80 cc [7], 26 cc [10] and 25 cc [9], using MATVs based on all pre-infusion ^18^F-FDG PET/CT-scans. These differences can be explained by the heterogeneity in used segmentation methods (Syngo-volume-counting-program, manually segmented, the relative SUV threshold 41% SUVmax, and the fixed threshold SUV 2.5 we used). In our recent publication comparing several segmentation methods, the application of the fixed threshold SUV 2.5 resulted in the highest median MATV, while the 41% SUVmax method led to the lowest median MATV, explaining our higher cut-off values [18]. The 41% SUVmax method is recommended by the EANM, but only for higher tumor-to-background values and homogenous tracer uptake [16]. For LBCL, several studies preferred other SUV thresholds due to known heterogeneity of this disease, leading to an underestimation of the tumor volume when 41% SUVmax method is used [19, 20]. This phenomenon has also been described by Dean et al. in the setting of CAR T-cell treatment for LBCL, resulting in their preference of manually segmented analyses above the 41% SUVmax method [6]. To maintain robustness and avoid the exclusion of tumor areas, a fixed threshold of SUV 2.5 was used in our research.

However, validation of our optimal cut-off point for MATV at baseline and pre-LD using the same segmentation method needs to be performed. Nevertheless, our results align with previous studies in terms of the survival benefit of reduced and controlled tumor volume resulting from effective bridging. Ultimately, a standardized and optimal segmentation method for LBCL is needed to reduce these variations between studies.

A limitation of these kind of studies including the current, is that bridging modalities were chosen according to the treating physician and not randomized. This could induce the occurrence of selection bias, as disease location, spread, and tumor volume could influence the choice of bridging regimen and thereby also the MATV dynamic. Additionally, performing detailed analyses on the response to bridging in combination with MATV-dynamics as well as other FDG PET characteristics would be of interest. Especially since Breen et al. already showed that other increased FDG PET characteristics, such as total lesion glycolysis (TLG), from pre-apheresis to pre-LD are associated with worse survival outcomes [11].

In conclusion, we demonstrate that r/r LBCL patients treated with CD19-directed CAR T-cell therapy with low baseline tumor volume measured using MATV, had a better TTP and OS and a lower incidence of severe CRS compared to high volume patients. In addition, effective reducing and controlling tumor volume during the bridging period in patients with a high baseline volume improved survival outcomes, providing rationale for the use of more aggressive bridging therapy regimens.

### Supplementary information

Supplementary File 1

Supplementary Tables

Supplementary Figure 1

## Data Availability

The data generated in this study are available upon request from the corresponding author.

## Code availability

R codes are available from the corresponding author on reasonable request.

## References

[1] Westin JR, Oluwole OO, Kersten MJ, Miklos DB, Perales M-A, Ghobadi A, et al. Survival with Axicabtagene Ciloleucel in Large B-Cell Lymphoma. N Engl J Med. 2023. 10.1056/NEJMOA2301665/SUPPL_FILE/NEJMOA2301665_DATA-SHARING.PDF. 10.1056/NEJMoa2301665 37272527

[2] Crump M, Neelapu SS, Farooq U, Van Den Neste E, Kuruvilla J, Westin J (2017). Outcomes in refractory diffuse large B-cell lymphoma: results from the international SCHOLAR-1 study. Blood.

[3] Locke FL, Ghobadi A, Jacobson CA, Miklos DB, Lekakis LJ, Oluwole OO (2019). Long-term safety and activity of axicabtagene ciloleucel in refractory large B-cell lymphoma (ZUMA-1): a single-arm, multicentre, phase 1-2 trial. Lancet Oncol.

[4] Roddie C, Neill L, Osborne W, Iyengar S, Tholouli E, Irvine D, et al. Effective bridging therapy can improve CD19 CAR-T outcomes while maintaining safety in patients with large B-cell lymphoma. Blood Adv. 2023. 10.1182/BLOODADVANCES.2022009019. 10.1182/bloodadvances.2022009019 PMC10300297 36724512

[5] Pinnix CC, Gunther JR, Dabaja BS, Strati P, Fang P, Hawkins MC (2020). Bridging therapy prior to axicabtagene ciloleucel for relapsed/refractory large B-cell lymphoma. Blood Adv.

[6] Dean EA, Mhaskar RS, Lu H, Mousa MS, Krivenko GS, Lazaryan A, et al. High metabolic tumor volume is associated with decreased efficacy of axicabtagene ciloleucel in large B-cell lymphoma. 2020. 10.1182/bloodadvances.2020001900. 10.1182/bloodadvances.2020001900 PMC7391155 32702097

[7] Galtier J, Vercellino L, Chartier L, Olivier P, Tabouret-Viaud C, Mesguich C (2023). Positron emission tomography-imaging assessment for guiding strategy in patients with relapsed/refractory large B-cell lymphoma receiving CAR T cells. Haematologica.

[8] Sjöholm T, Korenyushkin A, Gammelgård G, Sarén T, Lövgren T, Loskog A, et al. Whole body FDG PET/MR for progression free and overall survival prediction in patients with relapsed/refractory large B-cell lymphomas undergoing CAR T-cell therapy. Cancer Imaging 2022;22. 10.1186/S40644-022-00513-Y. 10.1186/s40644-022-00513-y PMC9793670 36575477

[9] Iacoboni G, Simó M, Villacampa G, Catalá E, Carpio C, Díaz-Lagares C (2021). Prognostic impact of total metabolic tumor volume in large B-cell lymphoma patients receiving CAR T-cell therapy. Ann Hematol.

[10] Hong R, Tan Su Yin E, Wang L, Zhao X, Zhou L, Wang G, et al. Tumor burden measured by 18F-FDG PET/CT in predicting efficacy and adverse effects of chimeric antigen receptor T-Cell Therapy in Non-Hodgkin Lymphoma. 2021;11. 10.3389/fonc.2021.713577. 10.3389/fonc.2021.713577 PMC8371710 34422666

[11] Breen WG, Young JR, Hathcock MA, Kowalchuk RO, Thorpe MP, Bansal R (2023). Metabolic PET/CT analysis of aggressive Non-Hodgkin lymphoma prior to Axicabtagene Ciloleucel CAR-T infusion: predictors of progressive disease, survival, and toxicity. Blood Cancer J.

[12] Bethge WA, Martus P, Schmitt M, Holtick U, Subklewe M, von Tresckow B (2022). GLA/DRST real-world outcome analysis of CAR T-cell therapies for large B-cell lymphoma in Germany. Blood.

[13] Kuhnl A, Roddie C, Kirkwood AA, Tholouli E, Menne T, Patel A (2022). A national service for delivering CD19 CAR-Tin large B-cell lymphoma – The UK real-world experience. Br J Haematol.

[14] Nastoupil LJ, Jain MD, Feng L, Spiegel JY, Ghobadi A, Lin Y (2020). Standard-of-Care Axicabtagene Ciloleucel for Relapsed or Refractory Large B-Cell Lymphoma: Results From the US Lymphoma CAR T Consortium. J Clin Oncol.

[15] Sidorenkov G, Nagel J, Meijer C, Duker JJ, Groen HJM, Halmos GB, et al. The OncoLifeS data-biobank for oncology: a comprehensive repository of clinical data, biological samples, and the patient’s perspective. J Transl Med. 2019;17. 10.1186/S12967-019-2122-X. 10.1186/s12967-019-2122-x PMC6857242 31727094

[16] Boellaard R, Delgado-Bolton R, Oyen WJG, Giammarile F, Tatsch K, Eschner W (2015). FDG PET/CT: EANM procedure guidelines for tumour imaging: version 2.0. Eur J Nucl Med Mol Imaging.

[17] Boellaard R, Oyen WJG, Hoekstra CJ, Hoekstra OS, Visser EP, Willemsen AT (2008). The Netherlands protocol for standardisation and quantification of FDG whole body PET studies in multi-centre trials. Eur J Nucl Med Mol Imaging.

[18] Keijzer K, Niezink AGH, de Boer JW, van Doesum JA, Noordzij W, van Meerten T (2023). Semi-automated 18F-FDG PET segmentation methods for tumor volume determination in Non-Hodgkin lymphoma patients: a literature review, implementation and multi-threshold evaluation. Comput Struct Biotechnol J.

[19] Eude F, Toledano MN, Vera P, Tilly H, Mihailescu SD, Becke S (2021). Reproducibility of baseline tumour metabolic volume measurements in diffuse large B-Cell Lymphoma: is there a superior method?. Metabolites.

[20] Ilyas H, Mikhaeel NG, Dunn JT, Rahman F, Møller H, Smith D, et al. Defining the optimal method for measuring baseline metabolic tumour volume in diffuse large B cell lymphoma. Eur J Nucl Med Mol Imaging. 2018;45. 10.1007/S00259-018-3953-Z. 10.1007/s00259-018-3953-z PMC5953976 29460024

[21] Barrington SF, Meignan M (2019). Time to prepare for risk adaptation in lymphoma by standardizing measurement of metabolic tumor burden. J Nucl Med.

[22] Cheson BD, Fisher RI, Barrington SF, Cavalli F, Schwartz LH, Zucca E (2014). Recommendations for initial evaluation, staging, and response assessment of hodgkin and non-hodgkin lymphoma: the lugano classification. J Clin Oncol.

[23] Lee DW, Santomasso BD, Locke FL, Ghobadi A, Turtle CJ, Brudno JN (2019). ASTCT consensus grading for cytokine release syndrome and neurologic toxicity associated with immune effector cells. Biol Blood Marrow Transplant.

[24] Wang J, Hu Y, Yang S, Wei G, Zhao X, Wu W (2019). Role of fluorodeoxyglucose positron emission tomography/computed tomography in predicting the adverse effects of chimeric antigen receptor T cell therapy in patients with non-hodgkin lymphoma. Biol Blood Marrow Transpl.

[25] Vercellino L, Di Blasi R, Kanoun S, Tessoulin B, Rossi C, D’Aveni-Piney M (2020). Predictive factors of early progression after CAR T-cell therapy in relapsed/refractory diffuse large B-cell lymphoma. Blood Adv.

[26] Cohen D, Luttwak E, Beyar-Katz O, Hazut Krauthammer S, Bar-On Y, Amit O (2022). [18F]FDG PET-CT in patients with DLBCL treated with CAR-T cell therapy: a practical approach of reporting pre- and post-treatment studies. Eur J Nucl Med Mol Imaging.

[27] Hubbeling H, Silverman EA, Michaud L, Tomas AA, Shouval R, Flynn J (2023). Bridging radiation rapidly and effectively cytoreduces high-risk relapsed/refractory aggressive B Cell lymphomas prior to chimeric antigen receptor T Cell Therapy. Transpl Cell Ther.

[28] Derlin T, Schultze-Florey C, Werner RA, Möhn N, Skripuletz T, David S (2021). 18F-FDG PET/CT of off-target lymphoid organs in CD19-targeting chimeric antigen receptor T-cell therapy for relapsed or refractory diffuse large B-cell lymphoma. Ann Nucl Med.

