# Supplementary material for: Reducing and controlling metabolic active tumor volume prior to CAR T-cell infusion can improve survival outcomes in patients with large B-cell lymphoma
Source: Blood Cancer J. 2024 Mar 7;14(1):41. doi: 10.1038/s41408-024-01022-w (PMC10917787; doi:10.1038/s41408-024-01022-w)
Supplement: Supplementary file 2 — Supplementary Tables [file 41408_2024_1022_MOESM2_ESM.docx]

|  | **TTP** | | **OS** | | **CRS** | | **ICANS** | |
| --- | --- | --- | --- | --- | --- | --- | --- | --- |
|  | **HR [95% CI]** | **P-value** | **HR [95% CI]** | **P-value** | **OR [95% CI]** | **P-value** | **OR [95% CI]** | **P-value** |
| LDH at screening (U/cl) | 1.22 [0.98 - 1.51] | 0.072 | 1.30 [1.03 - 1.55] | **0.028** | 1.11 [0.86 - 1.43] | 0.425 | 0.96 [0.73 - 1.26] | 0.757 |
| LDH pre-LD (U/cl) | 1.83 [1.35 - 2.47] | **< 0.001** | 1.80 [1.37 - 2.27] | **< 0.001** | 1.42 [0.92 - 2.18] | 0.116 | 0.97 [0.75 - 1.25] | 0.816 |
| Baseline MATV (L) | 1.68 [1.01 - 2.79] | **0.047** | 1.80 [1.09 - 2.82] | **0.021** | 1.58 [0.77 - 3.26] | 0.213 | 0.96 [0.45 - 2.06] | 0.917 |
| Pre-LD MATV (L) | 3.58 [1.78 - 7.17] | **< 0.001** | 4.30 [2.18 - 8.40] | **< 0.001** | 1.77 [0.62 - 5.10] | 0.287 | 1.84 [0.63 - 5.32] | 0.264 |

**Supplementary Table 1.** Additional univariable analysis for TTP*,* OS, CRS and ICANS

Abbreviations: CI, confidence intervals; CRS, cytokine release syndrome; HR, hazard ratios; ICANS, immune effector cell-associated neurotoxicity; L, liter; LDH, lactate dehydrogenase; pre-LD, pre-lymphodepleting; MATV, metabolic activated tumor volume; OR, odd ratios; OS, overall survival; TTP, Time to Progression; U/cl, units per centiliter

**Supplementary Table 2.** Patient, treatment and outcome characteristics per low- and high-MATV baseline patient groups (*n* = 74)

| **Characteristic** | **Low MATV baseline**  **(*n* = 37)** | **High MATV baseline**  **(*n* = 37)** | **P-value** |
| --- | --- | --- | --- |
| **Age in years**, median (range) | 62 (20 - 79) | 59 (27 - 79) | 0.213 |
| **Age > 65 years**, n (%) | 14 (37.8) | 10 (27.0) | 0.456 |
| **Gender, male**, n (%) | 20 (54.1) | 31 (83.8) | **0.012** |
| **Lymphoma histology, n (%)** |  |  | 0.783 |
| DLBCL | 20 (54.1) | 20 (54.1) |  |
| tFL | 8 (21.6) | 11 (29.7) |  |
| HGBCL DH/TH | 6 (16.2) | 4 (10.8) |  |
| HGBCL NOS | 3 (8.1) | 2 (5.4) |  |
| **ECOG PS, n (%)** |  |  | 0.354 |
| 0 | 28 (75.7) | 27 (73.0) |  |
| 1 | 9 (24.3) | 8 (21.6) |  |
| 2 | 0 (0) | 2 (5.4) |  |
| **Disease stage (at baseline), n (%)** |  |  | 0.344 |
| Stage I-II | 8 (21.6) | 4 (10.8) |  |
| Stage III-IV | 29 (78.4) | 33 (89.2) |  |
| **Nr. of extranodal sites, n (%)** |  |  | 0.624 |
| 0 | 13 (35.1) | 10 (27.0) |  |
| 1 | 14 (37.8) | 18 (48.6) |  |
| ≥2 | 10 (27.0) | 9 (24.3) |  |
| **LDH at screening > ULN**, n (%) | 17 (45.9) | 25 (67.6) | 0.094 |
| **LDH at screening**, median (IQR) | 245 (207 - 312) | 305 (241 - 550) | 0.008 |
| Missing, n (%) | 1 (2.7) | 1 (2.7) |  |
| **LDH pre-infusion > ULN**, n (%) | 6 (16.2) | 18 (48.6) | **0.006** |
| **LDH pre-infusion**, median (IQR) | 188 (168 - 240) | 237 (206 - 325) | **0.011** |
| **IPI, n (%)** |  |  | 0.852 |
| Low | 6 (16.2) | 9 (24.3) |  |
| Low-intermediate | 14 (37.8) | 12 (32.4) |  |
| High-intermediate | 14 (37.8) | 13 (35.1) |  |
| High | 2 (5.4) | 2 (5.4) |  |
| Missing | 1 (2.7) | 1 (2.7) |  |
| **Previous lines of therapy**, median (range) | 2 (1 - 6) | 2 (1 - 5) | 0.398 |
| **Primary refractory first line**, n (%) | 24 (64.9) | 27 (73.0) | 0.615 |
| **Primary refractory second line**, n (%) | 26 (70.3) | 27 (73.0) | 1 |
| N/A | 6 (16.2) | 4 (10.8) |  |
| **Days between apheresis and infusion**, median (IQR) | 31 (29 - 33) | 32 (28 - 35) | 0.222 |
| **Bridging therapy, n (%)** |  |  | **< 0.001** |
| No bridging | 21 (56.8) | 0 (0) |  |
| Radiotherapy | 11 (29.7) | 20 (54.1) |  |
| Systemic therapy | 3 (8.1) | 8 (21.6) |  |
| Combination | 2 (5.4) | 9 (24.3) |  |
| **Follow-up time since apheresis (months)**, median (IQR) | 23.4 (11.5 - 34.5) | 9.23 (6.0 - 14.7) | **< 0.001** |
| **Clinical response to CAR T-cell therapy, n (%)** |  |  |  |
| CR by last follow-up | 22 (59.5) | 16 (43.2) | 0.245 |
| Best ORR | 35 (94.6) | 29 (78.4) | 0.089 |
| **CRS grade, n (%)** |  |  | 0.007 |
| No CRS | 7 (18.9) | 5 (13.5) |  |
| 1 | 23 (62.2) | 11 (29.7) |  |
| 2 | 6 (16.2) | 20 (54.1) |  |
| 3 | 1 (2.7) | 1 (2.7) |  |
| **ICANS grade, n (%)** |  |  | 0.624 |
| No ICANS | 22 (59.5) | 16 (43.2) |  |
| 1 | 4 (10.8) | 8 (21.6) |  |
| 2 | 5 (13.5) | 7 (18.9) |  |
| 3 | 5 (13.5) | 5 (13.5) |  |
| 4 | 1 (2.7) | 1 (2.7) |  |

Abbreviations: CAR T-cell therapy, chimeric antigen T-cell therapy; CR, Complete Response; CRS, cytokine release syndrome; ICANS, immune effector cell-associated neurotoxicity syndrome; DLBCL, Diffuse Large B-cell lymphoma; ECOG PS, Eastern Cooperative Oncology Group performance status; HGBCL DH/TH, high-grade B-cell lymphoma double-hit/triple-hit; HGBCL NOS, high-grade B-cell lymphoma not otherwise specified; IPI, International Prognostic Index; IQR, interquartile range; N/A, not applicable; LDH, lactate dehydrogenase; MATV, metabolic activated tumor volume; ORR, Overall Response Rate; tFL, transformerd follicular lymphoma.

**Supplementary Table 3.** Patient, treatment and outcome characteristics per MATV risk group (*n* = 68)

| **Characteristic** | **Low MATV baseline**  **Low MATV pre-LD**  **(*n* = 28)** | **Low MATV baseline**  **High MATV pre-LD**  **(*n* = 4)** | **High MATV baseline**  **Low MATV pre-LD**  **(*n* = 19)** | **High MATV baseline**  **High MATV pre-LD**  **(*n* = 17)** | **P-value** |
| --- | --- | --- | --- | --- | --- |
| **Age in years**, median (range) | 61 (35 - 79) | 69 (20 - 70) | 58 (27 - 73) | 60 (34 - 79) | 0.635 |
| **Age > 65 years**, n (%) | 9 (32.1) | 3 (75.0) | 4 (21.1) | 5 (29.4) | 0.208 |
| **Gender, male**, n (%) | 13 (46.4) | 4 (100) | 14 (73.7) | 16 (94.1) | **0.003** |
| **Lymphoma histology**, n (%) |  |  |  |  | 0.663 |
| DLBCL | 14 (50.0) | 2 (50.0) | 11 (57.9) | 8 (47.1) |  |
| tFL | 7 (25.0) | 0 (0) | 5 (26.3) | 6 (35.3) |  |
| HGBCL DH/TH | 5 (17.9) | 1 (25.0) | 3 (15.8) | 1 (5.9) |  |
| HGBCL NOS | 2 (7.1) | 1 (25.0) | 0 (0) | 2 (11.8) |  |
| **ECOG PS,** n (%) |  |  |  |  | 0.132 |
| 0 | 24 (85.7) | 1 (25.0) | 13 (68.4) | 13 (76.5) |  |
| 1 | 4 (14.3) | 3 (75.0) | 5 (26.3) | 3 (17.6) |  |
| 2 | 0 (0) | 0 (0) | 1 (5.3) | 1 (5.9) |  |
| **Disease stage** **(at baseline),** n (%) |  |  |  |  | 0.684 |
| Stage I-II | 6 (21.4) | 1 (25.0) | 2 (10.5) | 2 (11.8) |  |
| Stage III-IV | 22 (78.6) | 3 (75.0) | 17 (89.5) | 15 (88.2) |  |
| **Nr. of extranodal sites,** n (%) |  |  |  |  | 0.767 |
| 0 | 11 (39.3) | 1 (25.0) | 7 (36.8) | 3 (17.6) |  |
| 1 | 10 (35.7) | 2 (50.0) | 7 (36.8) | 10 (58.8) |  |
| ≥2 | 7 (25.0) | 1 (25.0) | 5 (26.3) | 4 (23.5) |  |
| **LDH at screening > ULN**, n (%) | 14 (50.0) | 2 (50.0) | 10 (52.6) | 14 (82.4) | 0.093 |
| **LDH at screening**, median (IQR) | 257 (218 - 315) | 266 (210 - 405) | 272 (209 - 379) | 451 (290 - 635) | **0.002** |
| Missing, n (%) | 1 (3.6) |  |  | 1 (5.9) |  |
| **LDH pre-infusion > ULN**, n (%) | 5 (17.9) | 1 (25.0) | 7 (36.8) | 10 (58.8) | **0.043** |
| **LDH pre-infusion**, median (IQR) | 199 (171 - 240) | 168 (155 - 245) | 212 (196 - 269) | 315 (226 - 484) | **0.002** |
| **IPI**, n (%) |  |  |  |  | 0.933 |
| Low | 7 (25.0) | 1 (25.0) | 4 (21.1) | 2 (11.8) |  |
| Low-intermediate | 9 (32.1) | 1 (25.0) | 9 (47.4) | 5 (29.4) |  |
| High-intermediate | 10 (35.7) | 2 (50.0) | 5 (26.3) | 8 (47.1) |  |
| High | 1 (3.6) | 0 (0.0) | 1 (5.3) | 1 (5.9) |  |
| Missing | 1 (3.6) |  |  | 1 (5.9) |  |
| **Previous lines of therapy**, median (range) | 2 (2 - 5) | 2 (1 -2) | 2 (1- 5) | 2 (2 - 4) | 0.321 |
| **Primary refractory first line**, n (%) | 17 (60.7) | 3 (75.0) | 12 (63.2) | 14 (82.4) | 0.463 |
| **Primary refractory second line**, n (%) | 22 (78.6) | 3 (75.0) | 12 (63.2) | 15 (88.2) | 0.266 |
| N/A | 2 (7.1) | 1 (25.0) | 2 (10.5) | 1 (5.9) |  |
| **Days between apheresis and infusion**, median (IQR) | 31 (29 - 33) | 33 (30 - 35) | 32 (28 - 34) | 32 (30 - 35) | 0.701 |
| **Bridging therapy, n (%)** |  |  |  |  | **< 0.001** |
| No bridging | 13 (46.4) | 3 (75.0) | 0 (0) | 0 (0) |  |
| Radiotherapy | 11 (39.3) | 0 (0) | 13 (68.4) | 7 (41.2) |  |
| Systemic therapy | 3 (10.7) | 0 (0) | 4 (21.1) | 3 (17.6) |  |
| Combination | 1 (3.6) | 1 (25.0) | 2 (10.5) | 7 (41.2) |  |
| **Follow-up time since apheresis (months)**, median (IQR) | 24.1 (11.6 - 30.4) | 10.1 (7.3 - 20.3) | 9.8 (7.0 - 14.6) | 6.2 (3.7 - 10.7) | **< 0.001** |
| **Clinical response to CAR T-cell therapy**, n (%) |  |  |  |  |  |
| CR by last follow-up | 17 (60.7) | 2 (50.0) | 10 (52.6) | 5 (29.4) | 0.239 |
| Best ORR | 27 (96.4) | 4 (100) | 17 (89.5) | 11 (64.7) | **0.017** |
| **CRS grade**, n (%) |  |  |  |  | 0.077 |
| No CRS | 6 (21.4) | 0 (0) | 3 (15.8) | 2 (11.8) |  |
| 1 | 18 (64.3) | 3 (75.0) | 6 (31.6) | 5 (29.4) |  |
| 2 | 3 (10.7) | 1 (25.0) | 9 (47.4) | 10 (58.8) |  |
| 3 | 1 (3.6) | 0 (0) | 1 (5.3) | 0 (0) |  |
| **ICANS grade**, n (%) |  |  |  |  | 0.186 |
| No ICANS | 16 (57.1) | 2 (50.0) | 8 (42.1) | 8 (47.1) |  |
| 1 | 3 (10.7) | 1 (25.0) | 5 (26.3) | 2 (11.8) |  |
| 2 | 4 (14.3) | 0 (0) | 5 (26.3) | 2 (11.8) |  |
| 3 | 5 (17.9) | 0 (0) | 1 (5.3) | 4 (23.5) |  |
| 4 | 0 (0) | 1 (25.0) | 0 (0) | 1 (5.9) |  |

Abbreviations: CAR T-cell therapy, chimeric antigen T-cell therapy; CR, Complete Response; CRS, cytokine release syndrome; ICANS, immune effector cell-associated neurotoxicity syndrome; DLBCL, Diffuse Large B-cell lymphoma; ECOG PS, Eastern Cooperative Oncology Group performance status; HGBCL DH/TH, high-grade B-cell lymphoma double-hit/triple-hit; HGBCL NOS, high-grade B-cell lymphoma not otherwise specified; IPI, International Prognostic Index; IQR, interquartile range; N/A, not applicable; LDH, lactate dehydrogenase; MATV, metabolic activated tumor volume; ORR, Overall Response Rate; tFL, transformerd follicular lymphoma.
