# Supplementary material for: Reducing and controlling metabolic active tumor volume prior to CAR T-cell infusion can improve survival outcomes in patients with large B-cell lymphoma
Source: Blood Cancer J. 2024 Mar 7;14(1):41. doi: 10.1038/s41408-024-01022-w (PMC10917787; doi:10.1038/s41408-024-01022-w)
Supplement: Supplementary file 3 — Supplementary Figure 1 [file 41408_2024_1022_MOESM3_ESM.pdf]

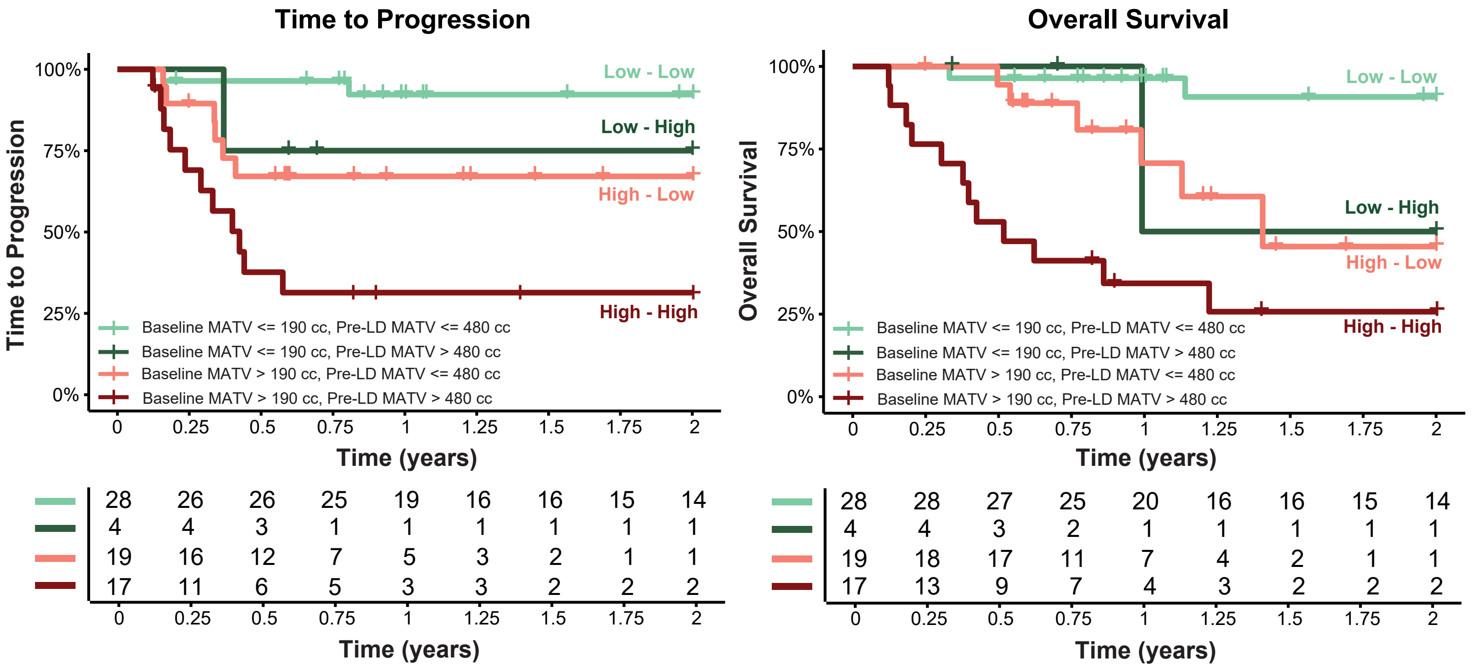

Supplementary Figure 1. Time to Progression and Overall Survival outcomes of the 4 patient risk groups with a low/high baseline MATV and a low/high pre-LD MATV (total *n* = 68)
